# Genome Analysis of Osteosarcoma Progression Samples Identifies *FGFR1* Overexpression as a Potential Treatment Target and *CHM* as a Candidate Tumor Suppressor Gene

**DOI:** 10.1371/journal.pone.0163859

**Published:** 2016-09-29

**Authors:** Tale Barøy, Chandra S. R. Chilamakuri, Susanne Lorenz, Jinchang Sun, Øyvind S. Bruland, Ola Myklebost, Leonardo A. Meza-Zepeda

**Affiliations:** 1 Department of Tumor Biology, Institute for Cancer Research, Norwegian Radium Hospital, Oslo University Hospital, NO-0310 Oslo, Norway; 2 Institute of Clinical Medicine, University of Oslo, Oslo, Norway; 3 Department of Oncology, Oslo University Hospital, Norwegian Radium Hospital, NO-0310 Oslo, Norway; 4 Norwegian Cancer Genomics Consortium, Oslo, Norway; 5 Genomics Core Facility, Department of Core Facilities, Institute for Cancer Research, Norwegian Radium Hospital, Oslo University Hospital, NO-0310 Oslo, Norway; Universite de Nantes, FRANCE

## Abstract

Osteosarcoma (OS) is the most common primary malignant tumor of bone, showing complex chromosomal rearrangements but with few known consistent changes. Deeper biological understanding is crucial to find new therapies to improve patient survival. We have sequenced the whole exome of two primary tumors (before and after chemotherapy), one metastatic tumor and a matched normal sample from two OS patients, to identify mutations involved in cancer biology. The metastatic samples were also RNA sequenced. By RNA sequencing we identified dysregulated expression levels of drug resistance- and apoptosis-related genes. Two fusion transcripts were identified in one patient (OS111); the first resulted in p53 inactivation by fusing the first exon of *TP53* to the fifth exon of *FAM45A*. The second fusion joined the two first exons of *FGFR1* to the second exon of *ZNF343*. Furthermore, *FGFR1* was amplified and highly expressed, representing a potential treatment target in this patient. Whole exome sequencing revealed large intertumor heterogeneity, with surprisingly few shared mutations. Careful evaluation and validation of the data sets revealed a number of artefacts, but one recurrent mutation was validated, a nonsense mutation in *CHM* (patient OS106), which also was the mutation with the highest expression frequency (53%). The second patient (OS111) had wild-type *CHM*, but a downregulated expression level. In a panel of 71 clinical samples, we confirmed significant low expression of *CHM* compared to the controls (p = 0.003). Furthermore, by analyzing public datasets, we identified a significant association between low expression and poor survival in two other cancer types. Together, these results suggest *CHM* as a candidate tumor suppressor gene that warrants further investigation.

## Background

Osteosarcoma (OS) is the most common primary malignant tumor of bone, with an overall incidence rate of 3.3 cases/million [1]. OS is highly aggressive [2, 3] and predominately affects children and adolescents [4]. Modern treatment protocols combine surgery, chemotherapy and sometimes radiotherapy, but the 5-year survival rate remains about 60–70% [5]. Recurrent or metastatic tumors are often multidrug resistant [6, 7], and the 20–25% of patients with metastatic disease at time of diagnosis have a 5-year survival rate at roughly 30% [8]. OS show complex chromosomal rearrangements with multiple gains and losses [9, 10], but few consistent changes are known. The clinical progress has been limited by a poor understanding of the massive, chaotic genetic events observed in this tumor type, and is further complicated by low incidence, limited material due to pre-surgery chemotherapy and routine decalcification of formalin-fixed paraffin-embedded tissue blocks.

By international collaboration, over 2,200 OS patients have been recruited to the EURAMOS-1 trial (European and American Osteosarcoma Studies) [11], the largest OS trial to date. The aim was to evaluate whether giving interferon to good responders or salvage chemotherapy (ifosfamide and etoposide) to the poor responders would improve the outcome compared to the standard treatment. Unfortunately, the event-free survival did not improve and severe side-effects increased [11]. Thus, deeper biological understanding seems crucial to develop new therapies to improve OS treatment.

The rapid advancement of high throughput sequencing allows comprehensive characterization of genomic changes, and by comparing spatial and temporal tumor biopsies the genetic basis for tumor progression, metastasis and treatment effects can be addressed. Furthermore, by sequencing OS tumors, mutations or translocations known in other cancer types may be identified, thereby opening the window for personalized cancer treatment for OS patients. The identification of new, potential treatment targets is especially important for those where the life-expectancy is low and the standard treatment options are few.

In this study, unique triplet progression samples consisting of primary tumors (before and after chemotherapy) and metastasis, of two OS patients have been studied in detail and compared to matched normal samples. We have sequenced the exomes of all samples and the transcriptome of the metastatic samples to study how these tumors relate, and to detect alterations associated with osteosarcoma biology, treatment, drug resistance and progression.

## Results

### Case descriptions

Both patients were included in the EURAMOS-1 trial at Oslo University Hospital and consented to our associated research project (approval ID from the Regional Ethics Committee of Southern Norway: S-06133). None of the patients experienced local recurrences during the course of their disease.

#### Case #1: OS106

**Fig 1:**
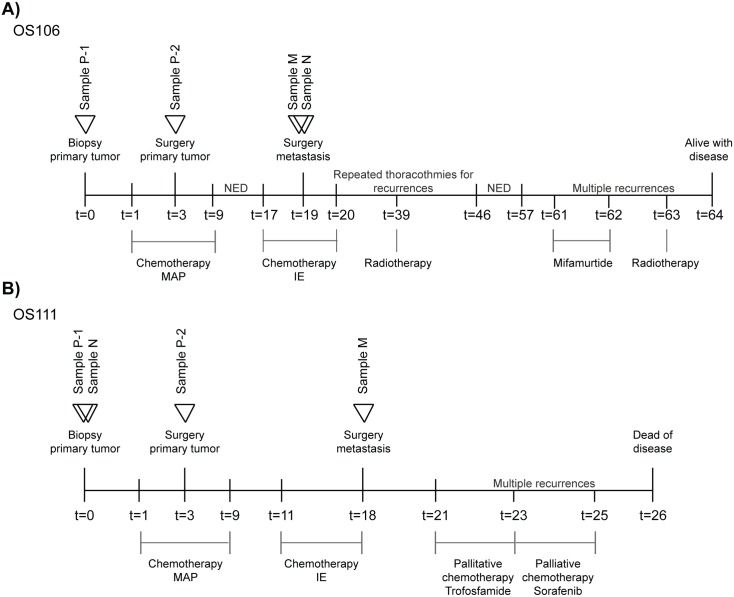
Schematic timeline of the disease and treatment courses of the two patients for (A): OS106 and (B): OS111. Time is shown in months. P-1: primary tumor treatment naïve, P-2: primary tumor treatment experienced. N: normal, M: metastasis, MAP: methotrexate, doxorubicin and cisplatin, IE: ifosfamide and etoposide, NED: No evidence of disease.

The first patient, a 16 years old male, hereafter referred to as OS106, presented a grade 4 primary OS in the proximal tibia without detectable metastases at diagnosis. The tumor was biopsied (sample P-1) before neoadjuvant treatment with chemotherapy (MAP: methotrexate, doxorubicin and cisplatin), followed by limb-salvage surgery after 2 months. The primary tumor (sample P-2) was removed with wide margins of only 2 mm towards the joint, but with a cut off of 20 mm as the shortest margin in normal soft tissues. The patient showed poor response to chemotherapy, defined in the trial as <90% necrosis [11] and was randomized to continue MAP postoperatively. Metastases in the lungs were discovered 17 months after the initial diagnosis. The patient then received a second line of chemotherapy (IE: ifosfamide and etoposide), followed by surgical removal of the lung metastases 2 months later (one of which is sample M). Surrounding healthy lung tissue, but distant from the tumor, was used as normal control (sample N). The patient progressed, and new lung metastases were detected. Stereotactic radiotherapy was given and four thoracotomies (two times each lung) were conducted to remove all macroscopic metastases. The patient had then no evidence of disease for 11 months, before metastases in the skull and costa were discovered. After treatment with the immunomodulator mifamurtide and radiotherapy, the patient is alive with disease (64 months after diagnosis). A schematic overview of the treatment timeline is presented in Fig 1A.

#### Case #2: OS111

The second patient, 37 years old female, hereafter referred to as OS111, presented a grade 4 primary OS in the distal femur without detectable metastases at diagnosis. The primary tumor was biopsied (sample P-1) and a blood sample (sample N) was obtained. The patient was enrolled in the EURAMOS-1 trial and received pre- and postoperative chemotherapy (MAP). The response to chemotherapy was good (≥90% necrosis), but the patient had severe side effects of methotrexate. The treatment courses were thus reduced and more delayed than originally scheduled. Two months after the start of chemotherapy, the primary tumor was removed by a limb-salvage procedure. The tumor was extending to the articular surface, but wide margins were obtained with 55 mm in the resected bone and 20 mm in soft tissue (sample P-2). Soon after completion of adjuvant chemotherapy, metastases were detected in both lungs. Chemotherapy (IE) was initiated, and the lung metastases were removed by surgery after 7 months (one of which is sample M). New metastatic lesions were discovered three months after the thoracotomy, and palliative chemotherapy (Ixoten/Trofosfamide followed by Nexavar/Sorafenib) was given. Metastases continued to appear and the patient succumbed 26 months after diagnosis. A schematic overview over the treatment timeline is presented in Fig 1B.

### Drug resistance is associated with transcriptional changes

The transcriptomes of both metastatic samples (OS106 M and OS111 M) were sequenced (RNA-seq), as well as four normal, primary osteoblast cultures that were used as controls. In total, OS106 had 1,934 downregulated transcripts (with a log2 fold reduction of <-2) and 8,089 upregulated transcripts (log2 fold increase of >2) compared to the osteoblast average. OS111 had a total of 2,140 downregulated transcripts and 9,350 upregulated transcripts. The transcriptome data and corresponding expression levels (as normalized read counts for each sample) are presented in S1 Table.

Both patients were considered to have acquired multidrug resistant disease. To identify possible causes of drug resistance, we investigated the expression of genes commonly associated with resistance to MAP. In particular, resistance is often caused by increased drug efflux caused by upregulation of members in the ATP-binding cassette (ABC) family of membrane transporters, downregulation of the genes *RFC1* and *DHFR*, mutations in *TOP2β* and upregulation of the *GSTP1* isoenzyme [12–25], but inhibition of apoptosis has also been reported to cause drug resistance [26–29].

**Fig 2:**
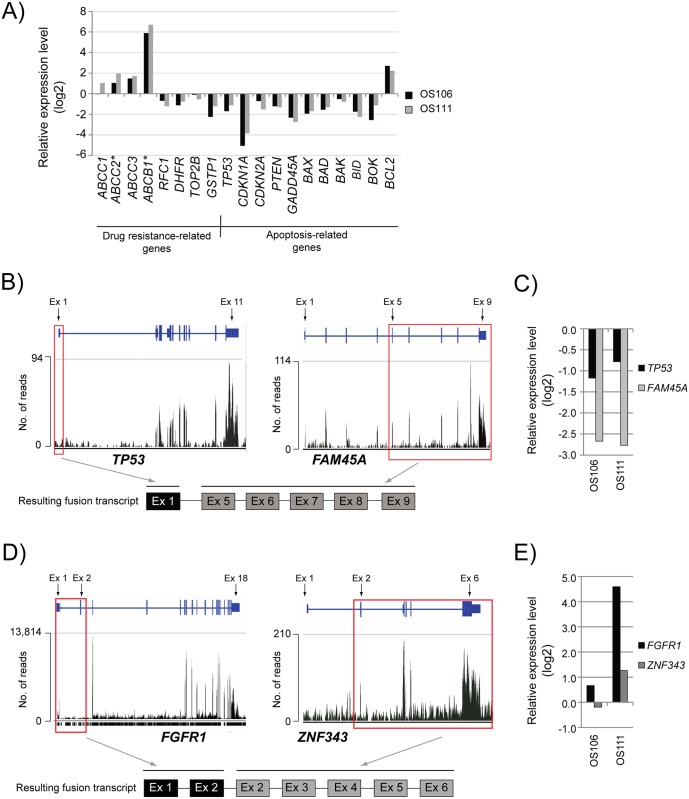
Transcriptome analysis of OS106 and OS111. **(A)** The relative expression level of known drug resistance-related genes and apoptosis-related genes. The values are shown as log2 of the expression level ratio, compared to the average of the four normal osteoblasts. Asterisk (*) indicates low number of reads in the osteoblasts. **(B)** The expression of all exons in *TP53* and *FAM45A*, respectively, shown as total number of reads in patient OS111. Red squares indicate the exons that are fused to form the resulting fusion transcript. ex: exon; no.: number **(C)** The relative expression level of *TP53* and *FAM45A*. The values are shown as log2 of the expression level ratio, compared to the average of the osteoblasts. The expression of all transcripts are included, both wild-type and aberrant. **(D)** The expression of all exons in *FGFR1* and *ZNF343*, respectively, shown as total number of reads in patient OS111. Red squares indicate the exons that are fused to form the resulting fusion transcript. **(E)** The relative expression level of *FGFR1* and *ZNF343*. The values are shown as log2 of the expression level ratio, compared to the average of the osteoblasts. The expression of all transcripts are included, both wild-type and aberrant.

The relative expression levels compared to the average of the osteoblasts are shown in Fig 2A. In OS111, the ABC transporters *ABCC1*, *ABCC2*, *ABCC3* and *ABCB1* (P-glycoprotein or multidrug resistance protein) were upregulated. The genes, except *ABCC1*, were also upregulated in OS106. However, upon inspection of the normalized read counts, it was apparent that the expression of both *ABCC2* and *ABCB1* was low in the controls and therefore the relative expression may be overestimated (indicated with an asterisk after the gene name in Fig 2A). The genes *RFC1* and *DHFR* were downregulated in both samples. There was no evidence of mutations in, or overexpression of, *TOP2β* in either of the samples, and *GSTP1* was rather found to be downregulated. We also identified dysregulation of several genes involved in apoptosis. *TP53* was downregulated in both patients, as were the tumor suppressors *CDKN1A*, *CDKN2A*, *PTEN* and *GADD45A*, which can be activated by both p53-dependent and -independent mechanisms. The pro-apoptotic genes *BAX*, *BAD*, *BAK*, *BID* and *BOK* were downregulated and the anti-apoptotic gene *BCL2* was upregulated (Fig 2A). Overall, gene expression profiles indicate an upregulation of drug resistance mechanisms and inhibition of apoptosis for both patients when compared to the normal controls.

In patient OS111, RNA-seq revealed a fusion transcript where the first exon of *TP53* was fused to the fifth exon of *FAM45A*. The expression profile of all exons in both genes, and an illustration of the corresponding fusion transcript are shown in Fig 2B (see S2 Table for fusion transcript breakpoint sequences). We did not find any evidence supporting the reciprocal fusion transcript. The expression levels (comparing the normalized read counts) of both *TP53* and *FAM45A* were downregulated in both patients compared to the average of the osteoblasts (Fig 2C). However, the fusion transcript was not highly expressed, as we identified a total of 11 paired-end reads spanning the breakpoint, thus additional mechanisms are likely to contribute to the downregulation of *TP53* in this patient. For patient OS106, we did not detect any aberrations of *TP53* that could explain the reduced expression level, indicating that the downregulation must be caused by mechanisms not detected using WES or RNA-seq.

### *FGFR1* is upregulated in OS111

A second fusion transcript was detected in OS111 (no fusion transcripts were detected in OS106), fusing *FGFR1* to *ZNF343*. *FGFR1* deregulation commonly occurs through gene amplification, point mutation or chromosomal translocations causing a constitutive receptor activation and ligand-independent signaling (reviewed in [30]). However, this particular fusion transcript contained only the first two exons of *FGFR1* fused to the second exon of *ZNF343* (Fig 2D). The first two exons of *FGFR1* encodes only the signal peptide [31], thus this particular fusion transcript does not result in an active FGFR1. We could not identify the reciprocal fusion transcript in our data, but the exons excluded in the fusion transcript (exons 3–18) have a substantial higher expression level than the two included in the fusion (exons 1–2) (Fig 2D). This could suggest high expression from a reciprocal fusion, but producing a transcript where the first exon of *ZNF343* is spliced out. On the other hand, many splice variants have been shown for FGFRs, including some lacking the signal peptide sequences [32], thus such transcripts could also originate from a wild-type (wt) allele. In OS111, the expression level of *FGFR1* was 24 times higher than the expression level in the osteoblasts (Fig 2E) (comparing normalized read counts).

Copy number analyses of all tumors were performed based on the whole exome data. For OS111, we found a large amplification on chromosome 8 including *FGFR1* in the P-1 and P-2 (in S1 Fig, copy number variation for all samples along chromosome 8:35,000,000–43,000,000 is shown). For sample M, of which the transcriptome data was obtained, there was no evidence of amplification, thus other mechanisms besides amplification must be causing the overexpression of *FGFR1* in the metastatic sample. Patient OS106 showed no amplification corresponding to the location of *FGFR1* (S1 Fig) and had an expression level comparable to the expression of the osteoblasts (1.6 fold) (Fig 2E).

The expression level of the fusion partner *ZNF343* was also upregulated in OS111 (2.4 fold of that in the osteoblasts), whereas the level in OS106 was at a similar level as the controls (0.8 fold) (Fig 2E). It is possible that the fusion between *FGFR1* and *ZNF343* in OS111 causes upregulation of *ZNF343*. This is further supported by the increase in expression of the exons included in the fusion transcript (exons 2–6), compared to exon 1 in *ZNF343* (Fig 2D).

### Whole exome sequencing analysis

**Fig 3:**
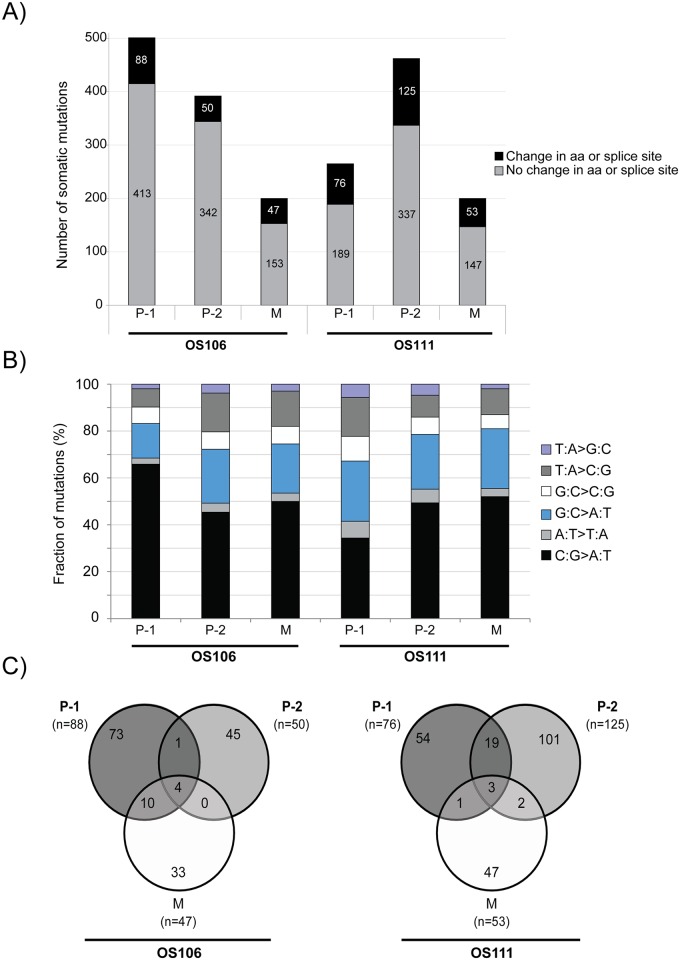
WES analysis of OS106 and OS111. **(A)** The total number of somatic mutations identified in each sample for both patients. Black represents nonsynonymous mutations in the coding sequence and/or mutations in the splice sites, whereas grey represents all other mutations. The respective numbers of mutations are indicated in the bars. aa: amino acids, P-1: primary tumor treatment naïve, P-2: primary tumor after treatment, M: metastasis. **(B)** The mutation spectrum showing the proportions of transitions and transversions in percent (adding up to 100%). **(C)** Venn diagram showing the distribution of mutations (only nonsynonymous mutations in the coding sequence and mutations in the splice sites) between the matched tumor samples in each patient. The total number of mutations in each tumor is shown in parenthesis.

The whole exomes of three progression samples (P-1, P-2 and M) and their corresponding normal sample (N) from both patients (OS106 and OS111) were sequenced (WES). Each tumor sample was compared to their respective normal sample using the validated Norwegian Cancer Genomics Consortium (NCGC, cancergenomics.no/en/) variant-calling pipeline [33], identifying between 200 and 500 somatic mutations per tumor (Fig 3A). Mutations in the introns were most frequent, followed by missense mutations, silent mutations and mutations in the untranslated regions (UTRs), respectively (see S2 Fig for an overview of the frequencies of different mutation types).

Analysis of the mutation spectra showed that C:G>A:T transversions were dominant for all samples, ranging from 34% to 66% frequency (Fig 3B). The second highest mutation rate was G:C>A:T transition, ranging from 15% to 26% frequency (Fig 3B). Both predominant mutation types have been associated with cisplatin DNA damage [34, 35]. In both patients the mutation spectrum of the treated sample (P-2) was more similar to the metastatic sample (M), than to the treatment naïve sample (P-1), further indicating that many of the mutations were induced by chemotherapy.

### WES reveals high intertumor heterogeneity

An overview of all somatic mutations and their corresponding coverage by WES and RNA-seq is shown in S3 Table. For further analysis, only the nonsynonymous mutations in the coding regions and the mutations in the splice sites were included. We focused on the recurrent mutations, present in at least two tumors in at least one of the patients. An overview over the number of mutations in each sample and the number of shared mutations between the tumors of each patient is shown in Fig 3C. However, some of the recurrent mutations were initially called from read sequences that mapped to more than one place in the genome with either identical or very high sequence similarity. The mutations called from such reads were discarded, even though they were reported in the COSMIC and/or the dbSNP databases. These were mutations in the genes *ARMC4* (p.D425Y), *MUC4* (p.A4217E, p.L4230P, p.H4205Q), *TIMM23B* (splice site, chr10: 51374435 A>G), *DDX11* (p.A607P, p.P368S), *CDC27* (p.F26S and p.L27P) and *FANCD2* (splice site, chr3: 10106408 C>T), demonstrating that care must be taken when evaluating mutation data. Two recurrent mutations (in *CHM* and *ZNF197)* were identified from unique read sequences. Both mutations were only present in patient OS106, and were validated by RNA-seq in the metastatic sample (expression frequency of 53% and 52% for the mutations in *CHM* and *ZNF197*, respectively). The mutation in *CHM* was further validated by targeted resequencing, but we were unable to design specific primers for the mutation in *ZNF197*. However, since the mutation in *ZNF197* results in one basic amino acid residue replacing another (p.K803R) we anticipate limited functional consequences. An overview of the coverage by WES, RNA-seq and targeted resequencing is shown in S4 Table.

### *CHM* is a candidate tumor suppressor gene

**Fig 4:**
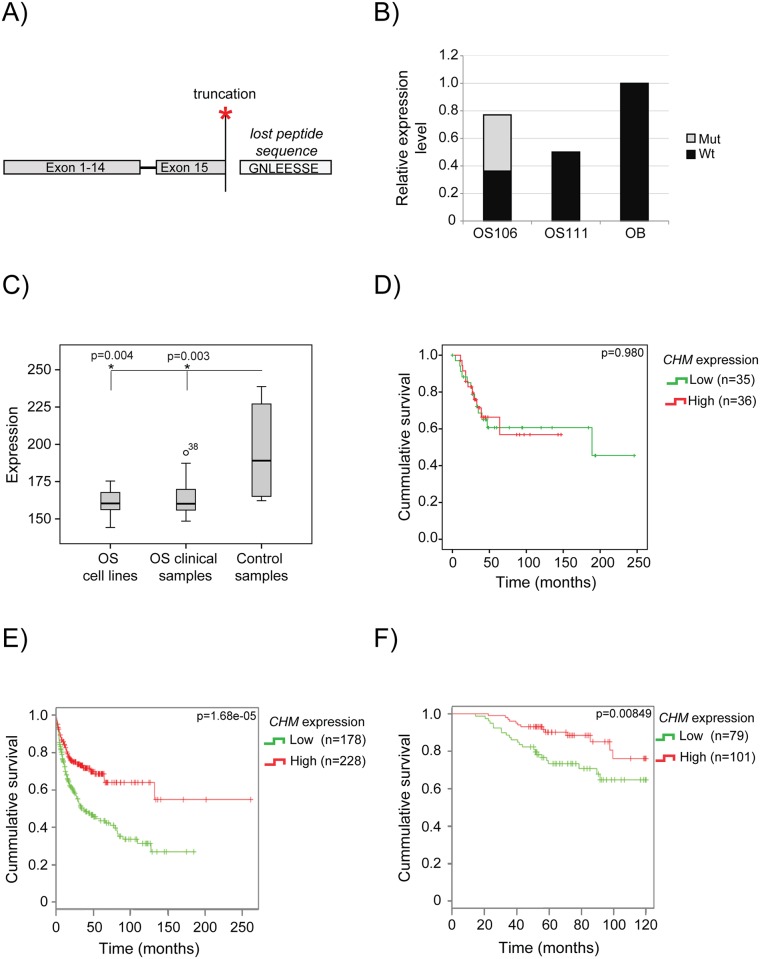
*CHM* analysis. **(A)** The identified nonsense mutation p.G646* in *CHM* results in the loss of the last eight amino acids. **(B)** The relative expression level of *CHM* in OS106 and OS111 compared to the average of the four osteoblasts. The nonsense mutation was only identified in OS106 at a frequency of 53% revealed by the RNA-seq (illustrated with grey bars). Mut: nonsense mutation, wt: wild-type. **C)** Expression level of *CHM in* osteosarcoma cell lines (n = 19), osteosarcoma clinical samples (n = 71) and normal control samples (n = 6). The difference was statistical significant with p-values of 0.004 and 0.003, for cell lines and clinical samples, respectively (Mann-Whitney U test) **(D)** Survival analysis of *CHM* expression in 71 clinical OS samples. The samples are sorted according to low (below median) or high (above the median) expression level. The difference was not significant with a p-value of 0.980 (Log Rank/Mantel-Cox test). **(E)** Survival plot obtained from DRUGSURV showing expression of *CHM* in patients with large diffuse B-cell lymphoma. Low expression was significantly associated with poor survival, p = 1.68e-05 (Chi-square 18.5 on 1 degree of freedom). **(F)** Survival plot obtained from DRUGSURV showing expression of *CHM* in patients with breast cancer. Low expression was significantly associated with poor survival, p = 8.49e-03 (Chi-square 6.9 on 1 degree of freedom).

The identified nonsense mutation in *CHM* (p.G646*), causing a truncation in the last exon (Fig 4A), was present in the treatment naïve primary tumor and in the metastasis of patient OS106. The expression level was similar to the average level in the four, normal osteoblasts, but 53% of the reads carried the mutation (Fig 4B). No mutation was detected in OS111, but the expression level of wt *CHM* was half that observed in osteoblasts (Fig 4B). We further compared the expression level of *CHM* in a panel of OS cell lines (n = 19) and OS clinical samples (n = 71) to control samples (four bone samples and two osteoblastic cultures (n = 6)). Gene expression profiling of these samples has previously been performed as a part of the EuroBoNeT network of excellence [36–38] (summary of the clinical data can be found in S5 Table). The expression level of *CHM* was significantly downregulated both in the cell lines and clinical samples compared to the control samples (Fig 4C). We also investigated whether the expression level of *CHM* in the clinical samples was correlated with survival. Of the 71 clinical samples, only one had an expression level above the median of the control samples. Thus, the survival analysis were performed using median split (Fig 4D), where the samples are stratified according to whether their respective expression level was above or below the median expression level of the clinical samples, and not the median level of the controls which was used in the expression level analysis (Fig 4C). No significant association between expression level and survival was found (Fig 4D). Using DRUGSURV, a publicly available database where expression profiling and clinical information from several studies are available [39], we investigated the expression of *CHM* in other cancer types. The top two hits were from studies on diffuse large B cell lymphoma [40] (GEO accession GSE10846) and breast cancer [41] (GEO accession GSE24450), and both showed that poor survival was significantly associated with low expression of *CHM* (Fig 4E and 4D, respectively). Together, these data suggest *CHM* as a candidate tumor suppressor gene in cancer, but the potential mechanism has to be further elucidated.

## Discussion

Osteosarcomas are rare tumors and it is difficult to obtain multiple sets of progression samples. By comparing matched progression tumors, novel insights into cancer biology may be revealed. Comparing the matched tumor progression genomes showed a high degree of intertumor heterogeneity. Other studies of matched tumor samples have shown a substantially higher level of concordance of shared mutations than what we found in this study, both by WGS [42, 43], WES [44] and targeted resequencing of smaller gene panels [45]. However, studies performed on multiple spatial biopsies in renal-cell carcinomas have identified large intratumor heterogeneity, with less than 40% shared mutations [46]. OS have exceptionally unstable genomes [47], which is probably causing the large heterogeneity seen in this study. In addition, two months of chemotherapy are separating samples P-1 and P-2, and further chemotherapy for the metastatic lesion. Not only have the tumors had time to acquire additional mutations, but also been under a strong selection pressure, which could increase the intertumor heterogeneity through *e*.*g*. branched evolution [48]. Not surprisingly, our data indicate that chemotherapy impacts the mutation burden in osteosarcoma. This also raises the question whether studies on treated tumors may include large amounts of mutational noise that is unrelated to the mechanisms of tumor development.

Both patients were considered to have acquired multidrug resistant disease. We found dysregulated expression levels of many of the genes reported to be involved in resistance to MAP [12–25]. Several of the ABC transporters were found to be upregulated, including *ABCB1*. Upregulation of *ABCB1*, also known as P-glycoprotein or multidrug resistance protein, is well known to confer drug resistance in OS [21, 23, 49–52]. *ABCB1* was highly upregulated in both patients, albeit the expression levels in the controls were low and consequently the relative expression level may be overestimated. In addition, we found several apoptosis-related genes to be dysregulated, also known to confer drug resistance [26–29]. Interestingly, we identified a fusion transcript joining the first exon of *TP53* to the fifth exon of *FAM45A* in OS111, most likely causing an inactivation of p53. Inactivation of *TP53* in osteosarcoma by rearrangements (with a hotspot in intron 1) has previously been reported by us and others [53–55], and may well be the cause of the fusion transcript in this patient, although intronic translocation sites cannot be confirmed by WES. However, also frequent trans-splicing of RNAs have been reported in OS cell lines [53]. The translocation in *TP53* appears to be an osteosarcoma-specific way to inactivate p53, and seems to be relatively frequent [53–55].

The second fusion transcript detected in OS111, joined exons 1–2 of *FGFR1* to exons 2–6 of *ZNF343*. The two first exons of *FGFR1* encode the signal peptide [31], which directs the location of *FGFR1* to the membrane. Thus, if translated, it is possible that the fusion protein is transported to the cell membrane. However, to our knowledge, there are no publications describing the function of *ZNF343*, thus making the potential function for such a fusion protein unclear. Interestingly, it has been shown that FGFR1 without the signal peptide accumulates in the cytosol and the nucleus [56], and that nuclear FGFR1 has been associated with invasive cancer cells [57–59]. The expression levels of the *FGFR1* exons excluded in the *FGFR1-ZNF343* fusion transcript (exons 3–18) were substantially higher than the ones included (exons 1–2). However, from our data we cannot conclude whether it contains the reciprocal fused allele (*ZNF343-FGFR1*) and produces a 5’ truncated *FGFR1* transcript, or a splice variant of a wt allele. Due to limited material, we were not able to investigate the subcellular localization of FGFR1 in the tumor samples.

FGFR1 is a high-priority therapeutic target, and there are both specific tyrosine kinase inhibitors and monoclonal antibodies targeting FGFRs [60]. *FGFR1* has been shown to be amplified in ~19% of OS tumors with poor response to chemotherapy [61]. Although OS111 had good response to the chemotherapy, drugs targeting FGFRs could have been of value for second-line treatment. Due to limited material, we were unable to establish cell lines or patient-derived xenografts, and no functional studies were possible. However, cancer cell lines with *FGFR1* amplification have been shown to be sensitive to an FGFR inhibitor (NVP-BGJ398) [62]. Interestingly, this was especially true for the osteosarcoma, breast and lung cancer cell lines [62]. Furthermore, pharmacological blockade of FGFR1 has also been shown to decrease lung metastases in an osteosarcoma animal model [63]. Thus, there are indications of possible treatment opportunities of OS patients with *FGFR1* amplification and/or overexpression.

Although still not strongly supported in OS, we propose *CHM* (choroideremia (Rab escort protein 1)) to be a candidate tumor suppressor gene. *CHM* encodes the protein Rab proteins geranylgeranyl transferase component A 1, hereafter referred to as REP-1. REP-1 recognizes newly synthesized Rab proteins and presents them to geranylgeranyl transferases for prenylation. Functioning as a chaperone, REP-1 keeps the hydrophobic geranylgeranylated Rab soluble and delivers it to the appropriate membrane [64], thought to rely on membrane receptors that recognize the complex between REP-1 and specific Rabs. Thus, REP-1 is important for the functionality of Rabs [65], which regulate intracellular vesicular transport [66]. Mutations in *CHM* are known to cause choroideremia; an X-linked form of progressive blindness caused by degeneration the retinal pigment epithelium and the two underlying cell layers. A second isoform called REP-2, encoded by the gene *CHML*, is believed to compensate for the loss of REP-1 in most human tissues, except the eye [67], which could explain why choroideremia patients seems otherwise unaffected. However, it has been shown that some Rab proteins are most efficiently prenylated by REP-1, *e*.*g*. Rab3A and Rab3D [67]. Furthermore, increased pH levels in lysosomes, reduced rates of proteolytic degradation and altered secretion of cytokines have been shown in monocytes and fibroblasts isolated from choroideremia patients compared to healthy controls [68]. This indicates that loss-of-function mutations in *CHM* is not necessarily fully compensated by REP-2, and could potentially affect intracellular transportation in cell types besides in the eye.

The identified nonsense mutation causes a truncation of the protein, losing the last 8 amino acids (of 654). To investigate the functional effects of the truncation was beyond the scope of this study. Three out of the eight amino acids are negatively charged (sequence: GNLEESSE), indicating a possible function for the C-terminal protein sequence. However, we found the expression level to be significantly downregulated in both OS cell lines and clinical samples compared to the control samples, which supports the assumption of an inhibitory role for *CHM* in osteosarcoma biology. We did not find a significant association between expression of *CHM* and survival in the osteosarcoma samples, which could be due to the overall low expression level in this sample cohort, but by using DRUGSURV, we found that low expression level of *CHM* in two other cancer types, large diffuse B cell lymphoma and breast cancer, were significantly associated with poor survival. It is possible that the level of *CHM* in OS is consistently low, and thus affects survival, but that almost all samples in our cohort had lower levels than the best stratum in the other comparisons. We propose, to our knowledge, for the first time a role for *CHM* in cancer biology, although more thorough studies are necessary to investigate the potential tumor suppressive function of REP-1.

## Materials and Methods

### Material

The patients included in the study consented to our research project in written. Our research project was approved by the Regional Ethics Committee of Southern Norway, with approval ID number: S-06133.

Tumor tissue was either surgical biopsy (P-1) or surgical specimens (P-2 and M). All were immediately snap-frozen in liquid nitrogen. Corresponding normal control samples (N) were either a blood sample (OS111) or healthy lung tissue (OS106).

Commercially available primary osteoblast cultures isolated from human femur and tibia of different donors (n = 4) (Cambrex BioScience) were used as normal control for the RNA-seq. The osteoblast cells were maintained in medium provided by the manufacturer, split when reaching 80% confluency and harvested when enough cells for RNA isolation were obtained.

### Nucleic acid isolation

DNA was isolated using Promega Wizard Genomic DNA purification isolation kit according to the manufacturer’s instructions and stored at 4°C. RNA was isolated using Qiagen miRNeasy isolation kit according to the manufacturer’s instructions and stored at -80°C. The integrity and quality of the DNA and RNA were assessed using an Agilent Technologies TapeStation and 2100 Bioanalyzer, respectively.

### Whole exome sequencing and analysis

The exome sequencing libraries of all tumor samples and matching normal was performed using the Agilent SureSelect All Exome v5 platform. One microgram of total genomic DNA was processed, as described in [69]. The libraries were sequenced paired-end (2 x 100 bp) on a HiSeq2500 (Illumina) using TruSeq SBS v3 chemistry. Real-time analysis and base calling were conducted by Illumina’s software packages HSC2.0.2/RTA1.17.21.3. Raw reads were processed using the Illumina CASAVA (v. 1.8.2) to demultiplex data and filter out the low-quality reads.

Initially reads were analyzed and quality checked using FastQC [70]. Alignment of reads to reference genome was performed by Novoalign [70]. Multiple mapping reads, PCR duplicates and not proper pair reads were removed using in-house scripts. Using GATK package, we performed local realignment around indels and base-quality recalibration [71]. Somatic single nucleotide variants were identified using MuTect [72]. Small InDel and raw copy number regions from exome were detected using VarScan 2 [73]. Copy number segments from raw data were identified using DNA copy package [70].

### Transcriptome sequencing and analysis

The RNA sequencing libraries were made using the Illumina TruSeq Stranded Total RNA with Ribo-Zero Gold kit, starting from one microgram high quality total RNA and processed according to the manufacturer’s instructions. The resulting libraries were sequenced paired end (2 x 100 bp) on a HiSeq2500.

Differential expression analysis from RNA-seq data performed by using DESeq package [74]. Fusion transcript analysis was performed as described in [53].

#### Data deposits

Because of Norwegian legal regulations, the ethical approval for this study and the consent signed by the patient, we are not able to deposit our sequencing dataset in a public repository. We will provide access to the data if requested by cancer researchers. For access, please contact the corresponding author Dr. Meza-Zepeda (leonardo.meza-zepeda@rr-research.no).

The gene expression data used in the *CHM* analysis can be retrieved from GEO. The accession numbers are GSE36004 (cell lines, osteoblasts and bone samples) and GSE30699 (clinical samples).

### Targeted resequencing

Validation of the mutations in *ZNF197* and *CHM* was performed by targeted resequencing. Primers were designed to amplify the mutations of interest with an amplicon length of ~140 bp. In addition, a tail complementary to the Barcoded Illumina Primers (Universal forward and indexed reverse primers) was added to the primers. The primer sequences are found in S6 Table.

The sequences were amplified using nested PCR. The procedure and reagents were identical to the set-up described in RainDance ThunderBolts Cancer Panel Assay Manual, with the exception of the custom-designed primers used in the 1^st^ PCR round and the annealing temperatures were set according to the melting temperatures of the respective primers. Furthermore, droplets were not generated, and thus the droplet stabilizer was not used.

The sequencing of the resulting libraries was performed on MiSeq (Illumina) using TruSeq SBS v3 chemistry. Analysis was performed using the PCR amplicon workflow using BWA for alignment and GATK for variant calling using the MiSeq Reporter software (Illumina).

## Supporting Information

S1 Fig Copy number variation. The copy number along chromosome 8:35,000,000–43,000,000 of each tumor sample, with emphasis on *FGFR1*.
(TIF)

S2 Fig Mutation annotation. An overview over the different mutations types for all tumor samples.
(TIF)

S1 Table Differential gene expression. The transcriptome data and corresponding gene expression levels (shown as normalized read counts) for each sample (tumors and controls).
(XLSX)

S2 Table Identified fusion transcripts. An overview over the identified fusion transcripts in patient OS111.
(XLSX)

S3 Table Expressed mutations. An overview over all identified somatic mutations and corresponding expression level in all samples.
(XLSX)

S4 Table Recurrent mutations. A detailed overview over the two recurrent mutations.
(XLSX)

S5 Table Clinical information. A summary of the clinical data of the 71 patients included in the *CHM* analysis.
(XLSX)

S6 Table Primer sequences. An overview over the primer sequences used for the targeted resequencing.
(XLSX)
