# Supplementary figures and images for: Genome Analysis of Osteosarcoma Progression Samples Identifies FGFR1 Overexpression as a Potential Treatment Target and CHM as a Candidate Tumor Suppressor Gene
Source: PLoS One. 2016 Sep 29;11(9):e0163859. doi: 10.1371/journal.pone.0163859 (PMC5042545; doi:10.1371/journal.pone.0163859)

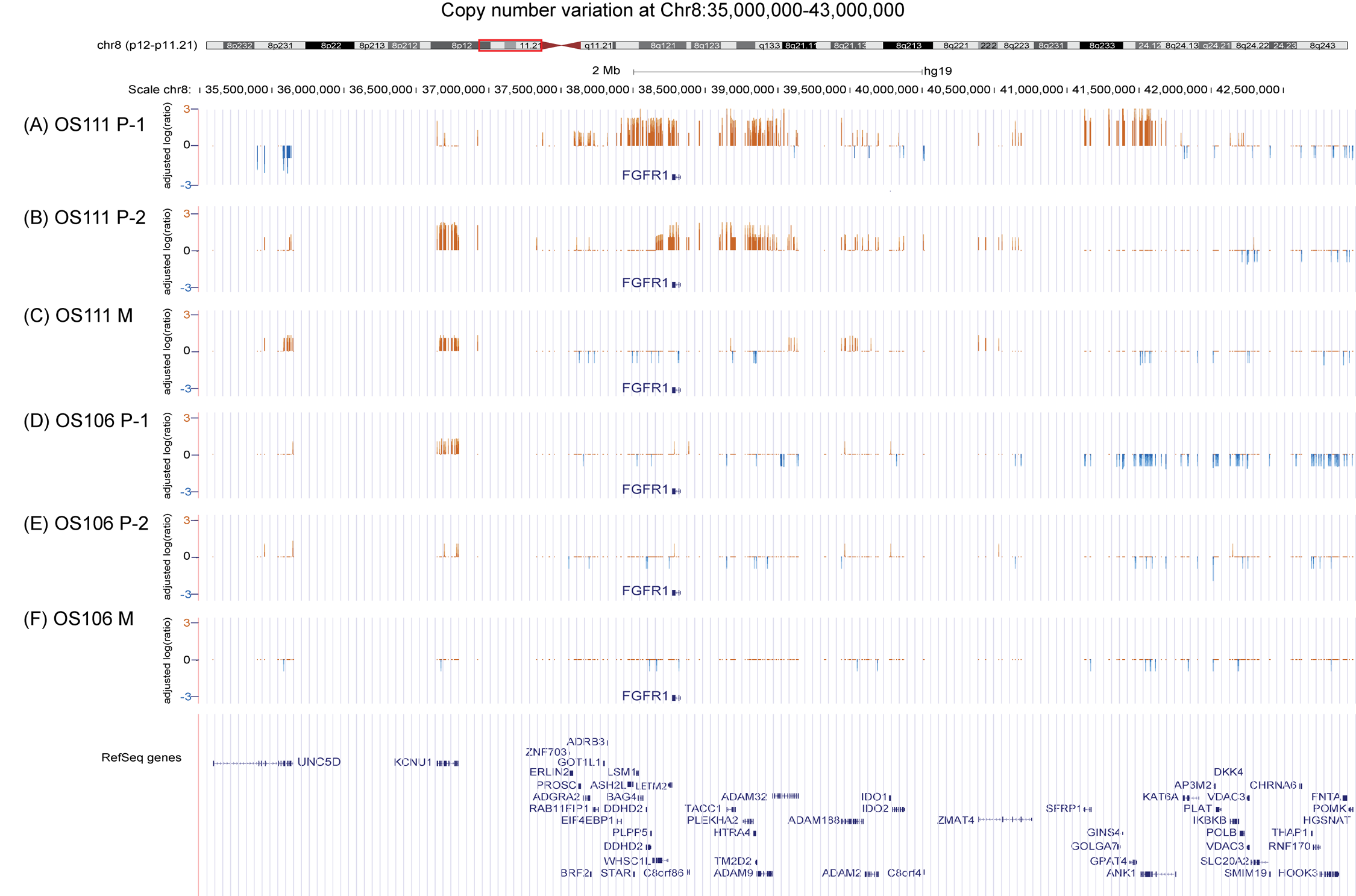

Supplement: S1 Fig — The copy number along chromosome 8:35,000,000–43,000,000 of each tumor sample, with emphasis on FGFR1. (TIF) [file pone.0163859.s001.tif]

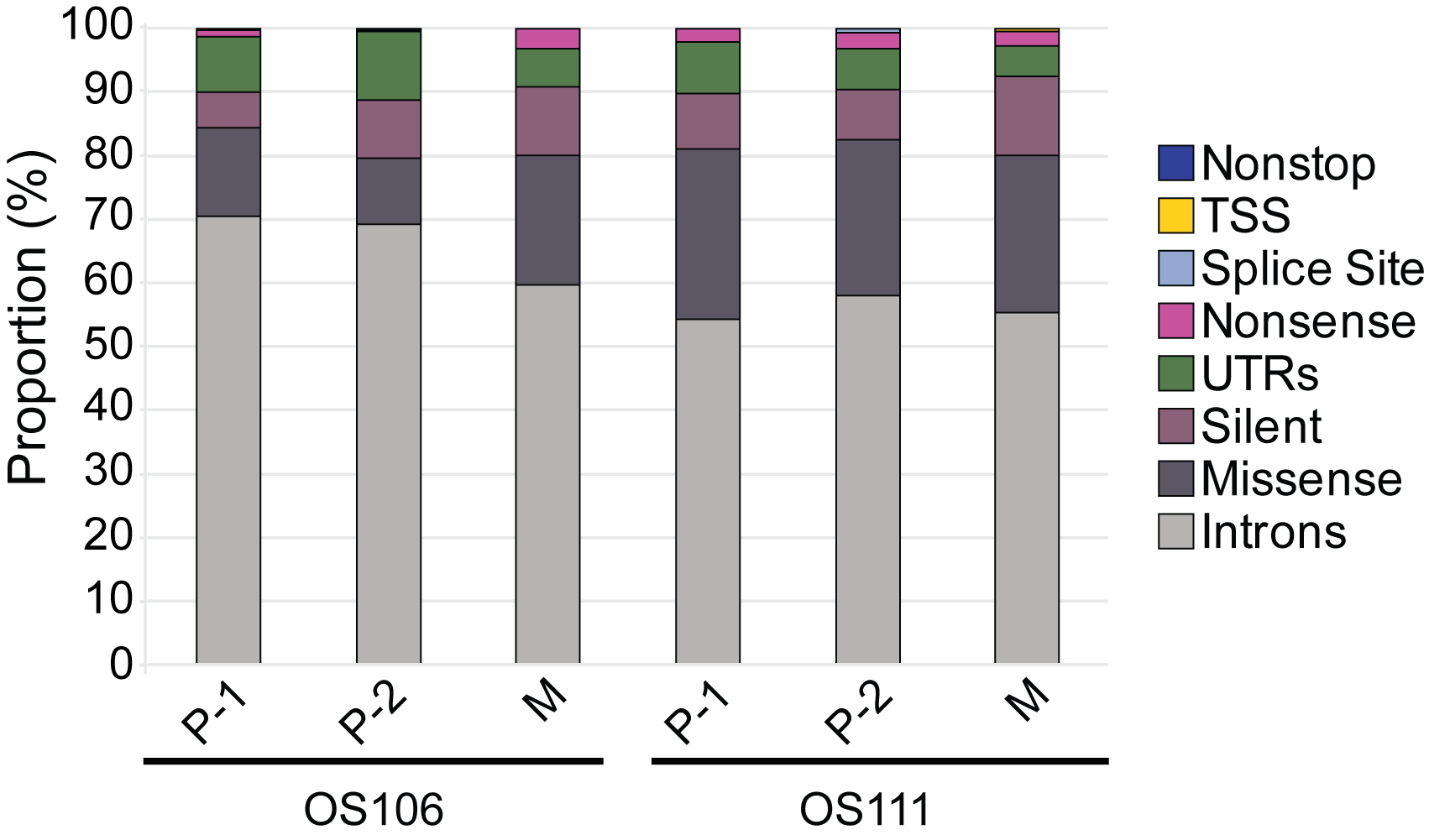

Supplement: S2 Fig — An overview over the different mutations types for all tumor samples. (TIF) [file pone.0163859.s002.tif]
